# A pilot study to determine the prevalence of HIV in persons presenting for care with selected conditions: preliminary results from the HIV in Europe study

**DOI:** 10.1186/1758-2652-13-S4-O16

**Published:** 2010-11-08

**Authors:** A Sönnerborg, A Mocroft, JD Lundgren, D Raben, J Gatell, A Vassilenko, V Hadziosmanovic, J Bergovac, H Sørensen, M Cusini, N Clumeck, B Gazzard, J Rockstroh, M Zuin, A D'Arminio Monforte

**Affiliations:** 1 Karolinska University Hospital, Department of Infectious Diseases, Stockholm, Sweden; 2 University College London Medical School, Research Department of Infection and Population He, London, UK; 3 Copenhagen HIV Programme, University of Copenhagen, Copenhagen, Denmark; 4 Hospital Clinic Barcelona, Infectious Diseases Unit, Barcelona, Spain; 5 Belarus State Medical University, Minsk, Belarus; 6 University of Sarajevo, Infectious Diseases Clinic, Sarajevo, Bosnia and Herzegovina; 7 University Hospital of Infectious Diseases, Zagreb, Croatia; 8 Bispebjerg Hospital, Århus, Denmark; 9 STD Centre, Dermatology Department, Milan, Italy; 10 Saint-Pierre University Hospital, Brussels, Belgium; 11 Chelsea Westminster Hospital, London, UK; 12 University of Bonn, Bonn, Germany; 13 San Paolo Hospital, Milan, Italy

## Purpose of the study

A pilot study was initiated in Autumn 2009 to better define which diseases have a HIV prevalence of >0.1 % as HIV testing of populations with a HIV prevalence above this has shown to be cost-effective. The preliminary results are reported here.

## Methods

A detailed questionnaire was completed for persons presenting with 8 different indicator diseases; sexually transmitted disease (STD), malignant lymphoma (LYM), cervical or anal cancer/dysplasia (CAN), herpes zoster (HER), ongoing mononucleosis-like illness (MON), unexplained leukocytopenia / thrombocytopenia lasting >4 weeks (CYT), and seborrheic dermatitis/exanthema (SEB).

## Results

**Figure 1:**
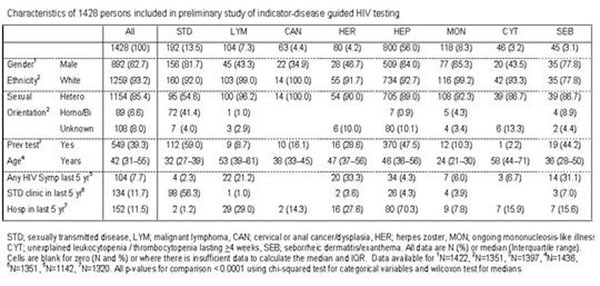


1482 persons have so far been included in the pilot phase by June 2010 from 29 surveys taking place in Austria, Belarus, Belgium, Bosnia, Croatia, Denmark, Germany, Italy, Poland, Spain, Sweden and Ukraine. Selected characteristics of the patients are shown in Figure 1. Almost 40% reported a previous HIV test; this was highest in the STD (59.0%), HEP (47.5%) and SEB (44.2%) groups. Median age was highest in the LYM group (53 years), and youngest in the MON group (24 years). 104 persons (7.7%) reported one of 5 specific HIV-related symptoms in the previous 5 years (mononucleosis, oral candidiasis, herpes zoster, unexplained leukocytopenia/thrombocytopenia or seborrheic dermatitis); these symptoms were highest in the HER (33.3%), SEB (31.1%) and LYM (21.2%) groups. 134 had visited an STD clinic in the previous 5 years including over half of the STD group (56.3%). 152 had been hospitalised in the previous 5 years; the highest proportion was seen in the HEP group (70.3%). Many of the planned surveys have shown difficult to implement because of reluctance towards introducing routine HIV testing among specialists who are not used to/worried about performing an HIV test. A number of persons have tested HIV-positive.

## Conclusions

The first results of this pilot study demonstrates the potential benefit of guided HIV testing of patients with selected indicator diseases, as a number of persons have been identified to be HIV-positive. A significant proportion of the persons had previously been hospitalised or reported HIV-associated symptoms but had not been tested. Physicians in some specialities are however reluctant to adopt this testing strategy.

